# Supplementary material for: Contributions of mirror-image hair cell orientation to mouse otolith organ and zebrafish neuromast function
Source: eLife. 2024 Nov 12;13:RP97674. doi: 10.7554/eLife.97674 (PMC11556791; doi:10.7554/eLife.97674)
Supplement: Supplementary file 1. [file elife-97674-supp1.docx]

|  | Type I | Type II | ALL |
| --- | --- | --- | --- |
| *Gpr156^del/+^* | 16/22 (73%) | 23/38 (60%) | 39/60 (65%) |
| *Gpr156^del/del^* | 20/29 (69%) | 34/46 (73%) | 54/75 (72%) |
| Age range (median) | P11-44 (P19) | P11-100 (P19) | P11-P100 (P18) |
| p value | 0.99 | 0.63 | 0.89 |

**Supplementary File 1. Percent of LES HCs with transduction**
