# Supplementary material for: Contributions of mirror-image hair cell orientation to mouse otolith organ and zebrafish neuromast function
Source: eLife. 2024 Nov 12;13:RP97674. doi: 10.7554/eLife.97674 (PMC11556791; doi:10.7554/eLife.97674)
Supplement: Supplementary file 2. — n.d., very fast component not detected. n/a, not available. [file elife-97674-supp2.docx]

|  |  |  |  | ***G(X) Boltzmann* parameters** | | | | ***Adaptation component tau at X-1/2*** | | | ***Adaptation component extent* at X-1/2*** | | | |
| --- | --- | --- | --- | --- | --- | --- | --- | --- | --- | --- | --- | --- | --- | --- |
| **HC Type** | **Genotype** | **n** | **Age (median)** | **G_max_, nS** | **10-90% OR, nm** | **X_1/2_, nm** | **dx, nm** | **τ_VF,_ ms (n)** | **τ_F,_ ms** | **τ_S_, ms** | **% decay** | **% A_VF_ (n)** | **% A_F_** | **% A_S_** |
|  |  |  |  |  |  |  |  |  |  |  |  |  |  |  |
| **Type I** | ***Gpr156^del/+^*** | 8 | P18-32 (P26) | 3.0 ± 0.4 | 780 ± 50 | 240 ± 20 | 180 ± 10 | 0.3 ± 0.1 (7) n.d. (1) | 5.1 ± 0.6 | 72 ± 12 | 54 ± 3 | 32 ± 5 (7) | 32 ± 7 | 36 ± 5 |
|  | ***Gpr156^del/del^*** | 5 | P19-25 (P22) | 2.5 ± 0.1 | 710 ± 70 | 230 ± 20 | 190 ± 30 | 0.3 ± 0.1 | 7.9 ± 1.6 | 112 ± 31 | 61 ± 3 | 40 ± 7 (5) | 28 ± 7 | 31 ± 3 |
|  |  |  |  |  |  |  |  |  |  |  |  |  |  |  |
|  | ***Statistics*** |  |  |  |  |  |  |  |  |  |  |  |  |  |
|  | **p value** |  |  | 0.15 | 0.37 | 0.7 | 0.83 | 0.55 | 0.15 | 0.18 | 0.12 | 0.34 | 0.72 | 0.45 |
|  | **NS power** |  |  | 0.21 | 0.13 | 0.07 | 0.05 | 0.09 | 0.42 | 0.25 | 0.34 | 0.14 | 0.06 | 0.11 |
|  |  |  |  |  |  |  |  |  |  |  |  |  |  |  |
|  | ***Gpr156^del/+^*** | **1** | P32 | 2.5 ± 0.2 | 670 ± 50 | 220 ± 10 | 160 ± 20 | 0.4 | 8.8 | 137 | 82 | 20 | 63 | 17 |
| **Type II** |  | 4 | P10-100 (P15) |  |  |  |  | n.d. | 9.1 ± 3.1 | 133 ± 88 | 73 ± 2 | 0 | 57 ± 3 | 43 ± 3 |
|  | ***Gpr156^del/del^*** | 1 | P22 | 2.2 ± 0.2 | 810 ± 50 | 280 ± 30 | 160 ± 20 | 0.52 | 4.4 | 47 | 75 | 26 | 41 | 33 |
|  |  | 4 | P14-44 (P22) |  |  |  |  | n.d. | 9.9 ± 5.7 | 117 ± 44 | 73 ± 7 | 0 | 52 ± 11 | 48 ± 11 |
|  |  |  |  |  |  |  |  |  |  |  |  |  |  |  |
|  | ***Statistics*** |  |  |  |  |  |  |  |  |  |  |  |  |  |
|  | **p value** |  |  | 0.39 | 0.08 | 0.27 | 0.79 | n/a | 0.91 | 0.88 | 0.97 | n/a | 0.66 | 0.66 |
|  | **NS power** |  |  | 0.12 | 0.43 | 0.17 | 0.06 | n/a | 0.05 | 0.05 | 0.05 | n/a | 0.07 | 0.07 |

**Supplementary File 2. Genotype comparisons of transduction and adaptation in LES HCs.** n.d., very fast component not detected. n/a, not available.

*Extent of adaptation component = 100 x [(Amplitude of component)/(sum of amplitudes of all components)]
