# Supplementary material for: Contributions of mirror-image hair cell orientation to mouse otolith organ and zebrafish neuromast function
Source: eLife. 2024 Nov 12;13:RP97674. doi: 10.7554/eLife.97674 (PMC11556791; doi:10.7554/eLife.97674)
Supplement: Supplementary file 3. — *estimated power of non-significant result. [file elife-97674-supp3.docx]

| **HC type** | **Genotype** | **n** | **Age (median)** | **C_m,_ pF** | **V_rest_,  mV** | **V_1/2_,  mV** | **Slope  factor, mV** | **R_in_  (MΩ)** | **G_max_/C_m,_  nS/pF** |
| --- | --- | --- | --- | --- | --- | --- | --- | --- | --- |
| **Type I** | *Gpr156^del/+^* | 9-13 | P15-34 (P19) | 5.8 ± 0.2 | -85 ± 1 | -83 ± 1 | 4.5 ± 0.3 | 46 ± 3 | 19 ± 2 |
|  | *Gpr156^del/del^* | 11-18 | P12-44 (P18) | 5.8 ± 0.3 | -86 ± 1 | -84 ± 1 | 4.8 ± 0.3 | 51 ± 3 | 24 ± 4 |
|  | p value |  |  | 0.84 | 0.84 | 0.57 | 0.53 | 0.37 | 0.22 |
|  | *NS power |  |  | 0.05 | 0.05 | 0.08 | 0.09 | 0.14 | 0.2 |
| **Type II** | *Gpr156^del/+^* | 24-29 | P11-47 (P18) | 4.7 ± 0.1 | -72 ± 1 | -31 ± 1 | 8.9 ± 0.5 | 598 ± 35 | 2.7 ± 0.2 |
|  | *Gpr156^del/del^* | 28-29 | P12-44 (P17) | 4.4 ± 0.1 | -73 ± 2 | -30 ± 1 | 9.0 ± 0.6 | 575 ± 41 | 3.2 ± 0.2 |
|  | p value |  |  | 0.14 | 0.71 | 0.48 | 0.85 | 0.67 | 0.11 |
|  | *NS power |  |  | 0.31 | 0.07 | 0.1 | 0.05 | 0.07 | 0.34 |

**Supplementary File 3. Genotype comparisons of electrical properties of Type I and II HCs in LES.** *estimated power of non-significant result
