# Supplementary material for: Contributions of mirror-image hair cell orientation to mouse otolith organ and zebrafish neuromast function
Source: eLife. 2024 Nov 12;13:RP97674. doi: 10.7554/eLife.97674 (PMC11556791; doi:10.7554/eLife.97674)
Supplement: Supplementary file 4. — Includes both cell types and all zones. n.d., very fast component not detected. [file elife-97674-supp4.docx]

|  |  |  | ***G(X) Boltzmann* parameters** | | | | ***Adaptation component tau at X_1/2_*** | | | ***Adaptation component extent at X_1/2_*** | | | |
| --- | --- | --- | --- | --- | --- | --- | --- | --- | --- | --- | --- | --- | --- |
| **Genotype** | **n** | **Age (median)** | **G_max_, nS** | **10-90% OR, nm** | **X_1/2_, nm** | **dx, nm** | **τ_VF,_ ms (n)** | **τ_F,_ ms** | **τ_S_, ms** | **% decay** | **% A_VF_** | **% A_F_** | **% A_S_** |
| ***Gpr156^del/+^*** | 23 | P10-100 (P18) | 3.0 ± 0.2 | 660 ± 40 | 200 ± 10 | 160 ± 10 | 0.3 ± 0.1 (14) n.d. (9) | 10.5 ± 1.8 | 152 ± 31 | 64 ± 2 | 24 ± 5 (14) n.d (9) | 39 ± 4 | 37 ± 3 |
| ***Gpr156^del/del^*** | 26 | P8-31 (P20) | 2.9 ± 0.2 | 720 ± 40 | 250 ± 20 | 160 ± 10 | 0.3 ± 0.3 (14) n.d. (12) | 7.8 ± 1.0 | 140 ± 21 | 68 ± 3 | 24 ± 5 (14) n.d (12) | 36 ± 4 | 40 ± 4 |
|  |  |  |  |  |  |  |  |  |  |  |  |  |  |
| ***Statistics*** |  |  |  |  |  |  |  |  |  |  |  |  |  |
| **p value** |  |  | 0.91 | 0.26 | 0.11 | 0.9 | 0.44 | 0.18 | 0.75 | 0.3 | 0.95 | 0.67 | 0.62 |
| **NS power** |  |  | 0.05 | 0.2 | 0.36 | 0.05 | 0.12 | 0.27 | 0.06 | 0.18 | 0.05 | 0.07 | 0.08 |

**Supplementary File 4. Genotype comparison of G(X) and adaptation parameters (all hair cells).** Includes both cell types and all zones**.** n.d. = very fast component not detected.
