# Supplementary material for: Contributions of mirror-image hair cell orientation to mouse otolith organ and zebrafish neuromast function
Source: eLife. 2024 Nov 12;13:RP97674. doi: 10.7554/eLife.97674 (PMC11556791; doi:10.7554/eLife.97674)
Supplement: Supplementary file 5. — *estimated power of non-significant result. [file elife-97674-supp5.docx]

| Genotype | n | Age range (median) | #  Transient | # Sustained | V_rest_, mV | R_in_, MΩ | I-threshold, pA |
| --- | --- | --- | --- | --- | --- | --- | --- |
| *Gpr156^del/+^* | 18 | P11-35 (P19) | 1 (5%) | 17 (94.5%) | -66 ± 1 | 614 ± 145 | 111 ± 27 |
| *Gpr156^del/del^* | 20 | P13-35 (P16) | 1 (5.0%) | 19 (95.0%) | -67 ± 1 | 323 ± 67 | 105 ± 20 |
| *Statistics* | | | | | | | |
| p value |  |  |  | 0.99 | 0.20 | 0.09 | 0.85 |
| *NS power |  |  |  |  | 0.25 | 0.43 | 0.05 |

**Supplementary File 5. Genotype comparison of excitability in LES afferents.** *estimated power of non-significant result.
